# Progressive Bounded Error Piecewise Linear Approximation with Resolution Reduction for Time Series Data Compression

**DOI:** 10.3390/s25010145

**Published:** 2024-12-29

**Authors:** Jeng-Wei Lin, Shih-wei Liao, Yu-Hung Tsai, Ching-Che Huang

**Affiliations:** 1 Department of Information Management, Tunghai University, Taichung 407224, Taiwan; jwlin@thu.edu.tw (J.-W.L.); g12490023@thu.edu.tw (Y.-H.T.); g12490013@thu.edu.tw (C.-C.H.); 2 Department of Computer Science and Information Engineering, National Taiwan University, Taipei 10617, Taiwan

**Keywords:** sensor data, time series, progressive data compression, hierarchical residual encoding, bounded error piecewise linear approximation, Swing-RR, PBEPLA-RR

## Abstract

Today, huge amounts of time series data are sensed continuously by AIoT devices, transmitted to edge nodes, and to data centers. It costs a lot of energy to transmit these data, store them, and process them. Data compression technologies are commonly used to reduce the data size and thus save energy. When a certain level of data accuracy is sacrificed, lossy compression technologies can achieve better compression ratios. However, different applications may have different requirements for data accuracy. Instead of keeping multiple compressed versions of a time series w.r.t. different error bounds, HIRE hierarchically maintains a tree, where the root records a constant function to approximate the whole time series, and each other node records a constant function to approximate a part of the residual function of its parent for a particular time period. To retrieve data w.r.t. a specific error bound, it traverses the tree from the root down to certain levels according to the requested error bound and aggregates the constant functions on the visited nodes to generate a new bounded error compressed version dynamically. However, the number of nodes to be visited is unknown before the tree traversal completes, and thus the data size of the new version. In this paper, a time series is progressively decomposed into multiple piecewise linear functions. The first function is an approximation of the original time series w.r.t. the largest error bound. The second function is an approximation of the residual function between the original time series and the first function w.r.t. the second largest error bound, and so forth. The sum of the first, second, …, and *m*-th functions is an approximation of the original time series w.r.t. the *m*-th error bound. For each iteration, Swing-RR is used to generate a Bounded Error Piecewise Linear Approximation (BEPLA). Resolution Reduction (RR) plays an important role. Eight real-world datasets are used to evaluate the proposed method. For each dataset, approximations w.r.t. three typical error bounds, 5%, 1%, and 0.5%, are requested. Three BEPLAs are generated accordingly, which can be summed up to form three approximations w.r.t. the three error bounds. For all datasets, the total data size of the three BEPLAs is almost the same with the size used to store just one version w.r.t. the smallest error bound and significantly smaller than the size used to keep three independent versions. The experiment result shows that the proposed method, referred to as PBEPLA-RR, can achieve very good compression ratios and provide multiple approximations w.r.t. different error bounds.

## 1. Introduction

Today, huge amounts of time series data are sensed continuously by AIoT (Artificial Intelligent + Internet of Things) devices, transmitted to edge nodes, and to data centers [1]. With these data, AIoT applications can understand our body, our car, and other objects on streets, the physical environment, machine statuses in factories, and so on. They can detect expected or unexpected changes and further react to certain conditions when necessary.

However, there are many challenges to realizing AIoT applications. One of them is the energy issue [2,3]. It costs a lot of energy to transmit the huge amount of data between system components via different networks, store them in disks or tapes temporarily or persistently, and process them periodically or in real-time.

Data compression is a common approach to not only reduce the data size and thus the network bandwidth and disk space [4,5,6,7,8,9,10,11,12] but also speed up the processing time with suitable data structures and algorithms [13,14,15]. In general, when lossless data compression technologies are used, the original data can be reconstructed without any loss of information. On the other hand, when a certain level of information loss is tolerated, lossy data compression technologies can usually achieve better compression ratios than lossless ones [4,5], such as the JPEG standard for images [16,17], and the H.263 standard for videos [18]. Sometimes, the differences between the reconstructed voice, pictures, and movies and the original data are undetectable by human eyes and ears. Sometimes, the differences are acceptable when people value other properties more important, for example, real-time access via a slow network connection.

For time series data compression, Bounded Error Piecewise Linear Approximation (BEPLA) is commonly used [6,7,8,9,10,11,12] when the difference between the original and the reconstructed time series at any time is smaller than or equal to a specific error bound. Here, the reconstructed time series is referred to as an approximation, or simply just a version in this article, of the original time series w.r.t. an error bound. In general, the larger the error bound, the smaller the compressed version, and the better the compression ratios.

However, different applications in different scenarios may have different data accuracy requirements. When a potential customer requests a copy of the original data for just a preview, the data owner prefers to return a rougher version. When the user uses a small mobile device, and thus a small screen, the resolution of an image is probably only 800 × 400 pixels, which is usually too small to display precise data. When the user is in a noisy environment, tiny variances in the data are usually ignored. On the other hand, some applications require high-quality data. For example, ECG identification uses ECG (electrocardiography) signals measured on smartwatches to identify a user [19]. When the network bandwidth varies with time, the acceptable quality of the received data may change accordingly [20].

When a request with an error bound comes, the system may always return the original time series, which is probably lossless compressed in advance, or a lossy version compressed w.r.t. the smallest possible error bound. This approach usually requires more network bandwidth. When necessary, the client performs precision reduction to the required data accuracy level.

The system may on-demand generate a compressed version of the original data according to the requested error bound. However, this approach burdens the system heavily. If the original data are lossless compressed, decompression is required before the system generates a new version. Otherwise, the system has to store the original data in uncompressed form.

The system may cache multiple versions of the original data, each of which is compressed w.r.t. a specific error bound. When requested, the system decides to generate a new version dynamically or just return the most suitable version in the cache. However, this approach requires more disk space to keep multiple versions w.r.t. different error bounds.

Progressive encoding is frequently used in multimedia streaming [17] and scientific research [21,22,23]. The original data are decomposed into multiple compressed components. The reconstructed approximation can be refined incrementally by adding more components. For exascale-scale scientific computing in climate, astronomy, and particle physics research, sophisticated data structures and data compression techniques are devised, such as precision-resolution trees for random data access [22] and bit-plane encoding for high-precision floating-point real numbers [23]. For time series data, HIRE was first proposed to maintain one data structure to generate multiple approximations w.r.t. different error bounds [24]. HIRE maintains a tree, where (1) a constant function is recorded on the root to approximate the whole original time series and thus a residual function between the two is derived, and (2) each other node records a constant function to approximate a part of the residual function of its parent for a particular time period recursively. When a request comes, HIRE traverses the tree from the root down to certain levels in many paths according to the requested error bound and aggregates the constant functions on the visited nodes to dynamically generate a new approximation. However, the number of nodes to be visited is unknown before the tree traversal completes, and so is the data size of the resultant approximation.

In this paper, we propose a novel progressive bounded error approximation algorithm for time series data, referred to as PBEPLA-RR (Progressive BEPLA with Resolution Reduction). Given a time series and *k* error bounds, PBEPLA-RR decomposes the time series into several BEPLAs. The first BEPLA is an approximation of the original time series w.r.t. the largest error bound. The second BEPLA is an approximation of the residual function between the original time series and the first BEPLA w.r.t. the second largest error bound, and so forth. It can be concluded that the sum of the first, second, …, and *m*-th BEPLAs is an approximation of the original time series w.r.t. the *m*-th error bound, where 1 ≤ *m* ≤ *k*. Iteratively, PBEPLA-RR adopts Swing-RR [12] to find *k* BEPLAs. Swing-RR is an efficient and effective algorithm to find a BEPLA for a time series. Resolution Reduction (RR) introduced in [12] plays an important role in each iteration, and the BEPLAs found by Swing-RR can be encoded by using very few bits. Hence, PBEPLA-RR can achieve very good performance while providing approximations w.r.t. multiple error bounds.

Swing-RR is very simple. The time and space complexities of Swing-RR are both optimal, *O*(*n*) and *O*(*d*), respectively, where *n* is the size of the time series and *d* is the maximal delay (in time ticks). When there are *k* error bounds, the time and space complexities of PBEPLA-RR are *O*(*kn*) and *O*(*d*), respectively. Since *k* is usually small, PBEPLA-RR is suitable for sensor nodes and edge nodes, which usually have very limited computing power, memory, network bandwidth, and battery life.

The remainder of this article is structured as follows: In Section 2, the concept of bounded error approximation and several BEPLA algorithms, including Swing-RR, are introduced. Then, a multiresolution scenario of bounded error time series compression is discussed. In Section 3, the concept of progressive bounded error approximation and its mathematical proof are given, and then the proposed algorithm, PBEPLA-RR. In Section 4, eight real world time series datasets are used to evaluate PBEPLA-RR. The experiment results are presented and discussed in depth. Finally, conclusions are given in Section 5 and also future works.

## 2. Related Works

### 2.1. Bounded Error Approximation

Given a time series *y*(*x*) = {*y*_1_, *y*_2_, *y*_3_, …, *y_n_*} and an error bound *ε*, another time series *y*′(*x*) = {*y*′_1_, *y*′_2_, *y*′_3_, …, *y*′*_n_*} is a bounded error approximation of *y*(*x*) when the following condition is satisfied.

|*y_t_* − *y*′*_t_*| ≤ *ε*, (1)

for 1 ≤ *t* ≤ *n*.

Under this restriction, Bounded Error Piecewise Linear Approximation (BEPLA) is widely used to approximate time series data. A BEPLA *S* = {*s*_1_, *s*_2_, *s*_3_, …, *s_c_*} consists of *c* non-overlapped line segments. Specifically, for a (time) tick *t* covered by a line segment *s_i_*, 1 ≤ *i* ≤ *c*, the value approximated by *S* (or *s_i_*) at tick *t* is

(2) $$S(t)=s_{i}.\mathrm{start}.y+\frac{s_{i}.\mathrm{stop}.y-s_{i}.\mathrm{start}.y}{s_{i}.\mathrm{stop}.x-s_{i}.\mathrm{start}.x}\times (t-s_{i}.\mathrm{start}.x),$$

where *s_i_*.start and *s_i_*.stop are the two endpoints of *s_i_*, and *s_i_*.start.x ≤ *t* ≤ *s_i_*.stop.x.

**Figure 1:**
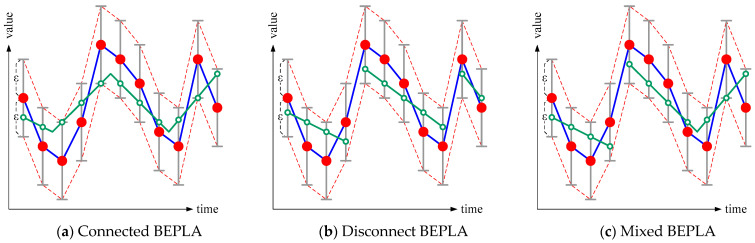
Concept of Bounded Error Piecewise Linear Approximation (BEPLA).

As shown in Figure 1, green line segments are used to approximate the original time series (red dots) with upper and lower error bounds. The distance between the approximated value (green circle) and the original value (red dot) at any tick is bounded by *ε*. I.e., green circles, which are intersections of green line segments and vertical time ticks, must be on the gray-bounded subranges.

In general, there are three types of BEPLA.

- For connected BEPLA, the line segments are connected;
- For disconnected BEPLA, the line segments are not connected;
- For mixed BEPLA, some line segments are connected, while some are not.

In connected BEPLA, the stop point of a line segment is used as the start point of its successive segment. I.e., (1) *s*_1_.start.x = 1, (2) *s*_*i*+1_.start = *s_i_*.stop, for 1 ≤ *i* < *c*, and (3) *s_c_*.stop.x = *n*. Thus, *S* can be recorded as {*s*_1_.start.y, (*s*_1_.length, *s*_1_.stop.y), (*s*_2_.length, *s*_2_.stop.y), …, (*s_c_*.length, *s_c_*.stop.y)}, where *s_i_*.length = *s_i_*.stop.x − *s_i_*.start.x, for 1 ≤ *i* ≤ *c*.

On the other hand, in disconnected BEPLA, a new line segment always starts one tick after its previous segment stops. I.e., *s*_*i*+1_.start.x = *s_i_*.stop.x + 1, for 1 ≤ *i* < *c*. Thus, *S* can be expressed as {(*s*_1_.start.y, *s*_1_.length, *s*_1_.stop.y), (*s*_2_.start.y, *s*_2_.length, *s*_2_.stop.y), …, (*s_c_*.start.y, *s_c_*.length, *s_c_*.stop.y)}.

In the literature, many BEPLA algorithms have been proposed. Some are used to generate connected BEPLA [6,7,8], while some are for disconnected BEPLA [9]. In general, connected BEPLA algorithms use fewer bits to encode a line segment than disconnected ones. However, since the start point of a new segment is always the stop point of its immediate previous segment, connected BEPLA algorithms use more segments than disconnected ones. Mixed BEPLA algorithms [10,11] choose connected or disconnected segments by dynamic programming techniques and achieve better compression ratios than connected and disconnected BEPLA algorithms.

For multi-dimensional time series data, each dimension is usually considered independently when BEPLA is adopted.

### 2.2. Swing-RR

**Figure 2:**
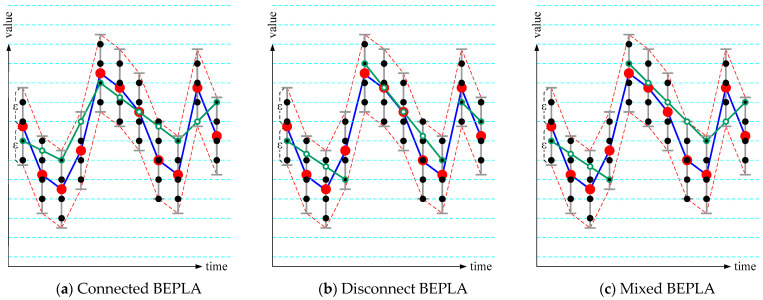
Concept of BEPLA with Resolution Reduction (RR).

In [12], the concept of Resolution Reduction (RR) was introduced. The two endpoints of line segments are restricted and thus can be encoded as small integers rather than floating-point real numbers. As shown in Figure 2, the two endpoints of segments are chosen from only black dots. The authors note that the approximated value (green circles), rather than the endpoints of segments, are not necessarily on black dots.

The number of bits used to encode the approximated value, i.e., *s_k_*.start.y and *s_k_*.stop.y, is called *resolution*. Given an error bound *ε*, the minimal resolution is given as the following equation, where *R* is the whole possible value range of the time series and *ε*/*R* is the relative error bound.

*r_min_* (*ε*/*R*) = ⌈−log_2_(*ε*/*R*) − 1⌉. (3)

Minimal resolutions for common relative error bounds are listed in Table 1. When *r* is used as the resolution, *r* ≥ *r_min_* (*ε*/*R*), the whole range *R* is divided into 2*^r^* blocks, and thus there are 2*^r^* block centers, represented as the horizontal dashed cyan lines in Figure 2. Black dots are intersections of horizontal dashed cyan lines and gray-bounded subranges. When *r* increases, there will be more black dots in the gray-bounded subranges. However, the experiment in [12] shows that the number of line segments does not decrease much. It depends more on the time series itself. On the other hand, the reduction from 32-bit floating-point real numbers to *r*-bit unsigned integers plays a significant role in data compression.

Based on this concept, Swing-RR was proposed in [12], which is simple and has optimal time and space complexities, *O*(*n*) and *O*(*d*), respectively, where *n* is the size of the time series and *d* is the maximal delay (in ticks), i.e., the maximal length of a line segment. The experiment results showed that Swing-RR effectively reduces the size of the original time series when minimal resolution is used.

### 2.3. Multiresolution Time Series Compression

In real words, different applications in different scenarios may have different data accuracy requirements [21,22,23,24]. There are several approaches. The first approach is usually referred to as ***one-size-fit-all***. When a request comes, the system may always return the original time series, which is probably lossless compressed in advance. Lossless data compressors usually achieve lower compression ratios than lossy data compressors. As a result, this approach requires larger storage space and higher network bandwidth. However, the system only compresses the time series once. The requesting node decompresses the received data and performs precision reduction to the desired quality level. There is a variant for the ***one-size-fit-all*** approach. The system may also keep a lossy compressed version w.r.t. the minimal possible error bound and return it always for requests with larger error bounds. This version is usually smaller than the lossless compressed one.

Another approach is to compress the original data w.r.t. different error bounds independently, and thus many versions are generated and kept in the system. When the system is requested, the nearest version satisfying the requested error bound is returned. For example, when the requested relative error bound is 3%, the version w.r.t. 1% error bound is chosen among versions w.r.t. 5%, 1%, and 0.5% error bounds. However, the size of the version w.r.t. 1% error bound is usually larger than the version w.r.t. 3% error bound. Thus, higher network bandwidth is required. When necessary, the requesting node performs precision reduction. Otherwise, the system may generate a new version w.r.t. the requested error bound on demand. However, the system load increases. The original data has to be kept uncompressed to avoid frequent decompression. The new version could be cached over a period of time for access in the near future.

The third is to adopt progressive compression [17,21,22,23], where the approximation could be refined incrementally when more data are received. It is wildly used in variable bit rate (VBR) audio and/or video streaming, such as JPEG 2000 [17]. However, in VBR streaming, data size (or bit rate) is the goal to meet, rather than data precision. Recently, sophisticated data structures and data compression techniques have been devised, such as precision-resolution trees for random data access [22] and bit-plane encoding for high-precision floating-point real numbers [23] in exascale scientific research, including climate, astronomy, and particle physics experiments and simulations.

For bounded error time series applications, HIRE was proposed in 2023 [24] that uses piecewise constant functions recorded in nodes of a tree in many levels to progressively represent the original time series. The root records a constant function to approximate the whole original time series. Then, the residual function between the two is derived and partitioned into smaller time periods. Similarly, each other node records a constant function to approximate a part of its parent’s residual function and computes its own residual function. For any period in the time series, a constant function could be derived by a pooling function, such as Mean(), Midrank(), Median(), or Random(), which outputs the mean, average of the minimal and maximal, median, or a random one from the period, respectively. By recursively partitioning, pooling, and residualizing, many constant functions, each of which covers a smaller period of its parent, are derived and organized hierarchically as nodes in a tree; as well, concatenation of children of a node is the corresponding residual function. This recursive process continues, and thus the tree deepens until the corresponding residual functions of all leaf nodes are smaller than or equal to the smallest error bound.

When a request comes with an error bound, the nodes for the specified time period are top-down traversed from the root, and at the same time the constant functions on the visited nodes are retrieved and aggregated until the residual functions are smaller than or equal to the given error bound.

The depth of the tree is *O*(log *n*), where *n* is the size of the time series. However, the final depth of the tree is somehow unpredictable before the compression finishes and so is the compression time. Similarly, for decompression with an error bound, the nodes that should be visited and their depths are unknown before the tree traverse completes, and so is the data size of the returned approximation.

## 3. Methods

In this section, the authors first introduce the concept of progressive bounded error approximation of a time series adopted in this paper and its mathematical proof. Then, the authors propose PBEPLA-RR that generates *k* BEPLAs based on a modified Swing-RR when *k* error bounds are specified. Pseudocodes of algorithms ***PBEPLA-RR*** and ***Approximate*** for compression and decompression follow. Given a required error bound, the latter ***Approximate*** reconstructs an approximation from the resultant *k* BEPLAs.

### 3.1. Progressive Bounded Error Approximation

As shown in Figure 1 and Figure 2, in typical bounded error approximation, an approximation *y*′(*x*) of an original time series *y*(*x*) is between *y*(*x*) − *ε*_1_ and *y*(*x*) + *ε*_1_ for any time tick, given an error bound *ε*_1_. The green line that approximates the blue lines is bounded by the two red dashed lines.

**Figure 3:**
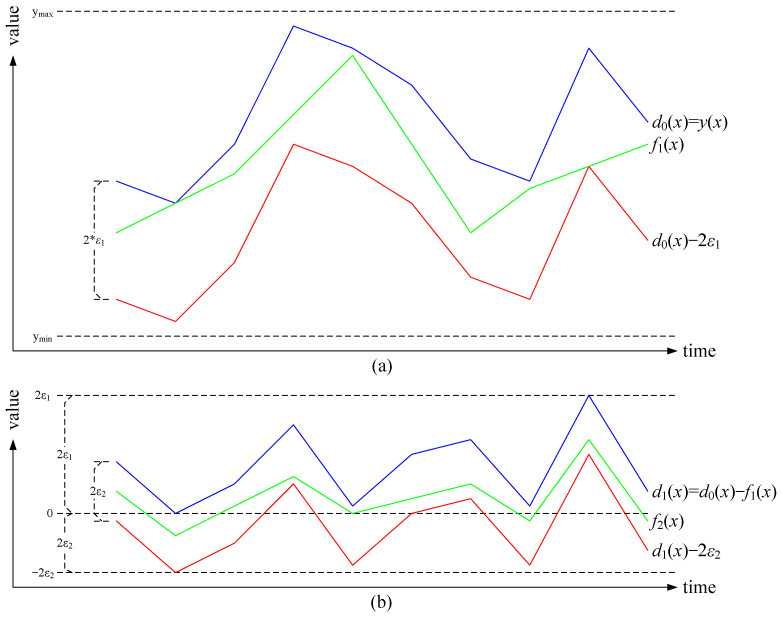
Concept of the Progressive Bounded Error Approximation. (**a**) *f*_1_(*x*) is an approximation of *d*_0_(*x*) between *d*_0_(*x*) − 2 × *ε*_1_ and *d*_0_(*x*), where *d*_0_(*x*) = *y*(*x*). (**b**) *f*_2_(*x*) is an approximation of *d*_1_(*x*) between *d*_1_(*x*) − 2 × *ε*_2_ and *d*_1_(*x*), where *d*_1_(*x*) = *d*_0_(*x*) − *f*_1_(*x*).

In progressive bounded error approximation, a different approximation between *y*(*x*) − 2 × *ε*_1_ and *y*(*x*) is used. As shown in Figure 3a, *f*_1_(*x*) is between *y*(*x*) − 2 × *ε*_1_ and *y*(*x*). It is trivial that the residual function *d*_1_(*x*) = *y*(*x*) − *f*_1_(*x*) is between 0 and 2 × *ε*_1_.

In other words, *y*(*x*) is decomposed into two bounded functions, *f*_1_(*x*) and *d*_1_(*x*), where *f*_1_(*x*) is an approximation between *y*(*x*) − 2 × *ε*_1_ and *y*(*x*), and *d*_1_(*x*) is the residual function between 0 and 2 × *ε*_1_, as shown in Figure 3b. When the second bound error *ε*_2_ is applied, *d*_1_(*x*) is further decomposed into two additional bounded functions, *f*_2_(*x*) and *d*_2_(*x*), where *f*_2_(*x*) is an approximation between *d*_1_(*x*) − 2 × *ε*_2_ and *d*_1_(*x*), and *d*_2_(*x*) is the residual function between 0 and 2 × *ε*_2_; and so are *d*_2_(*x*), *d*_3_(*x*), …, *d*_*k*−1_(*x*) recursively, when more error bounds, *ε*_3_, …, *ε_k_*, are applied.

The following proof shows that *f*_1_(*x*) + *f*_2_(*x*) + … + *f_m_*(*x*) + *ε_m_* is bounded between *y*(*x*) − *ε_m_* and *y*(*x*) + *ε_m_*, where 1 ≤ *m* ≤ *k*. I.e., it is a bounded error approximation of *y*(*x*), and the error bound is *ε_m_*.

Given a time series *y*(*x*) and a set of *k* error bounds, *ε*_1_, *ε*_2_, …, *ε_k_*, the goal is to find a set of bounded functions, *f*_1_(*x*) and *d*_1_(*x*), *f*_2_(*x*) and *d*_2_(*x*), …, and *f_k_*(*x*) and *d_k_*(*x*) recursively, satisfying the following conditions.

*d*_0_(*x*) = *y*(*x*), (4)

0 ≤ *d*_1_(*x*) = *d*_0_(*x*) − *f*_1_(*x*) ≤ 2 × *ε*_1_, (5)

…

0 ≤ *d_k_*(*x*) = *d_k_*_−1_(*x*) − *f_k_*(*x*) ≤ 2 × *ε_k_*, (6)

where *d*_0_(*x*) is defined to simplify the proof. It is trivial that *f_i_*(*x*) is an approximation of *d_i_*_−1_(*x*) between *d_i_*_−1_(*x*) − 2 × *ε_i_* and *d_i_*_−1_(*x*), for 1 ≤ *i* ≤ *k*, referred to Figure 3a,b; it is also easy to verify that for 1 ≤ *i* ≤ *k*,

−*ε_i_* ≤ *d_i_*_−1_(*x*) − (*f_i_*(*x*) + *ε_i_*) ≤ *ε_i_*. (7)

*F_m_*(*x*) is defined as Equation (8), where 1 ≤ *m* ≤ *k*.

*F_m_*(*x*) = *f*_1_(*x*) + *f*_2_(*x*) + … + *f_m_*(*x*). (8)

Since *f_i_*(*x*) equals to *d_i_*_−1_(*x*) − *d_i_*(*x*), 1 ≤ *i* ≤ *k*,

*F_m_*(*x*) = (*d*_0_(*x*) − *d*_1_(*x*)) + (*d*_1_(*x*) − *d*_2_(*x*)) + … + (*d_m_*_−1_(*x*) − *d_m_*(*x*))

=*d*_0_(*x*) − *d_m_*(*x*) = *y*(*x*) − *d_m_*(*x*) (9)

Thus,

*y*(*x*) − *F_m_*(*x*) = *d_m_*(*x*). (10)

Since 0 ≤ *d_m_*(*x*) ≤ 2*ε_m_*,

−*ε_m_* ≤ *d_m_*(*x*) − *ε_m_* = *y*(*x*) − *F_m_*(*x*) − *ε_m_* = *y*(*x*) − (*F_m_*(*x*) + *ε_m_*) ≤ *ε_m_*. (11)

*Y_m_*(*x*) is defined as Equation (12). According to Equation (11), *Y_m_*(*x*) is between *y*(*x*) − *ε_m_* and *y*(*x*) + *ε_m_*, and thus a bounded error approximation of *y*(*x*).

*Y_m_*(*x*) = *F_m_*(*x*) + *ε_m_* = *f*_1_(*x*) + *f*_2_(*x*) + … + *f_m_*(*x*) + *ε_m_*. (12)

### 3.2. Progressive BEPLA

As aforementioned, given an error bound, many BEPLA algorithms have been proposed to generate a BEPLA of a time series. Since Swing-RR [12] is simple and effective and thus suitable in sensor nodes and edge nodes, it is adopted and modified when progressive bounded error approximation is considered.

As shown in Figure 2, for typical BEPLA, at any time tick *t*, the black dots are on the subrange between *y*(*t*) − *ε* and *y*(*t*) + *ε*. However, the upper bound in Progressive BEPLA is *y*(*t*), and the lower bound is *y*(*t*) − 2 × *ε*, as shown in Figure 3a. As well, in typical BEPLA, the whole range should at least contain values from ***Min***(*y*(*x*) − *ε*) to ***Max***(*y*(*x*) + *ε*). On the other hand, in Progressive BEPLA, the whole range for the first error bound *ε*_1_ should at least contain values from ***Min***(*y*(*x*) − 2 × *ε*_1_) to ***Max***(*y*(*x*)). I.e., *y_min_* ≤ ***Min***(*y*(*x*) − 2 × *ε*_1_) ≤ ***Max***(*y*(*x*)) ≤ *y_miax_*. For the other error bound *ε_i_*, the range is from −2 × *ε_i_*_−1_ to 2 × *ε_i_*, as shown in Figure 3b, and the actual relative error bound is *ε_i_*/(2 × (*ε_i_* + *ε_i_*_−1_)), which is significantly larger than *ε_i_*/(*y_miax_* − *y_min_*). As a result, Swing-RR can adopt lower resolution and use fewer bits to encode the endpoints of line segments.

Algorithm 1 shows the pseudocode of ***PBEPLA-RR***. Given a time series *Y*[1…*n*], the value of which ranges between *min* and *max*, *k* error bounds *ε*[1…*k*], *k* resolutions *r*[1…*k*], one for each error bound, and the maximal delay, which limits the maximal length of line segments, ***PBEPLA-RR*** iteratively computes a BEPLA by the modified ***Swing-RR***. For every error bound, a BEPLA is derived, then an approximation by reconstructing the BEPLA, and finally the residual function by subtracting the approximation from *Y*[1…*n*]. The authors note that the initial values of *min* and *max* are most likely predefined and dependent on the data source rather than the minimal and maximal values of *Y*, respectively.
**Algorithm 1 *PBEPLA-RR*** (*Y*[1…*n*], *min*, *max*, *k*, *ε*[1…*k*], *r*[1…*k*], *delay*)
Input:    *Y*[1…*n*]: the time series to be compressed
      *min*, *max*: the range of possible values in *Y*
      *k*: the number of error bounds
      *ε*[1…*k*]: the *k* error bounds
      *r*[1…*k*]: *r*[*i*] is the resolution for ***Swing-RR*** to generate *BEPLA*[*i*]
      *delay*: the maximal delay for ***Swing-RR*** to generate all *BEPLAs*
Output: *BEPLA*[1…*k*]: the resultant *k* BEPLAs to approximate *Y*
      In the array, *BEPLA*[*i*] is a BEPLA to represent *f_i_*(*x*)
1: **begin**2:   *BEPLA*[1] := ***Swing_RR*** (*Y*[1…*n*], *min*, *max*, *ε*[1], *r*[1], *delay*)3:   *Y*[1…*n*] := *Y*[1…*n*] − ***Reconstruct*** (*BEPLA*[1])4:   **for** *i* := 2 to *k* **do**5:      *min* = −2*ε[*i*]6:      *max* = 2*ε[*i* − 1]7:      *BEPLA*[*i*] := ***Swing_RR*** (*Y*[1…*n*], *min*, *max*, *ε*[*i*], *r*[*i*], *delay*)8:      *Y*[1…*n*] := *Y*[1…*n*] − ***Reconstruct*** (*BEPLA*[*i*])9:   **end for**10:    **return** *BEPLA*[1…*k*]11:  **end**

The time and space complexities of ***Swing-RR*** are *O*(*n*) and *O*(*d*), respectively, where *n* and *d* are the number of data points and maximal delay [12]. When *k* error bounds are applied, the time complexity of ***PBEPLA-RR*** is *O*(*kn*). It is not uncommon that *k* is small. With proper implementation, the space complexity of ***PBEPLA-RR*** remains *O*(*d*).

**Figure 4:**
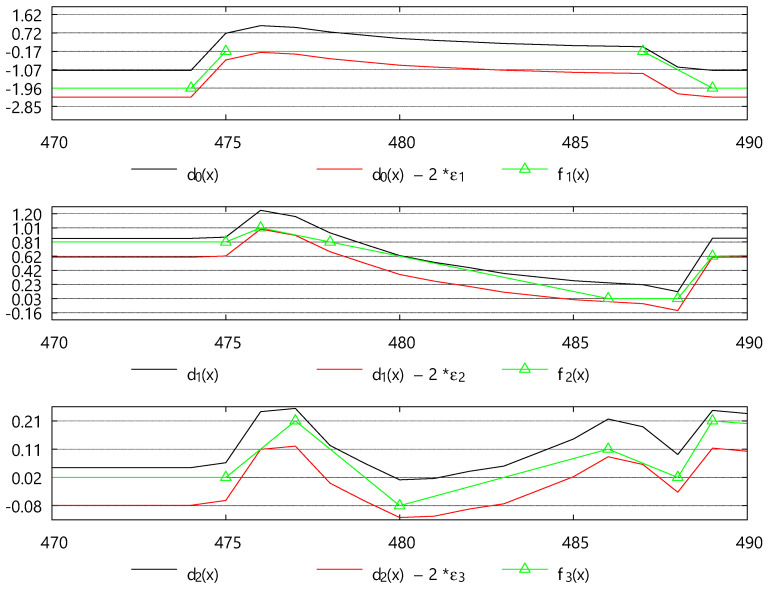
The three connected BEPLAs, *f*_1_(*x*), *f*_2_(*x*), and *f*_3_(*x*), generated by ***PBEPLA-RR*** for dataset Cricket_X, where *d*_0_(*x*) = *y*(*x*), *d*_1_(*x*) = *d*_0_(*x*) − *f*_1_(*x*), and *d*_2_(*x*) = *d*_1_(*x*) − *f*_2_(*x*).

Figure 4 shows the three connected BEPLAs generated by the modified ***Swing-RR*** in ***PBEPLA-RR*** for dataset Cricket_X, selected from the UCR time series classification archive [1]. The three error bounds are 5%, 1%, and 0.5%, respectively, and the resolutions for each error bound are 4, 3, and 2, respectively. The authors note that the actual relative error bounds for the second and third ***Swing-RR*** invocations are 1%/(2 × (1% + 5%)) = 8.3% and 0.5%/(2 × (0.5% + 1%)) = 16.7%, respectively. As a result, for each error bound, there are 2^4^, 2^3^, and 2^2^ blocks, and thus 2^4^, 2^3^, and 2^2^ block centers, which are represented as the horizontal dashed lines.

As expected, the endpoints of all line segments are on the horizontal dashed lines. This restriction may force ***Swing-RR*** to use more line segments. However, endpoint selection of line segments in transient periods is usually more restricted by the data themselves than Resolution Reduction.

### 3.3. Reconstruction from Progressive BEPLA

Given a required error bound ε*, algorithm ***Approximate*** returns a time series based on the *k* BEPLAs generated by algorithm ***PBEPLA-RR***, as shown in Algorithm 2.

**Algorithm 2 *Approximate*** (*k*, *ε*[1…*k*], *BEPLA*[1…*k*], ε*)
Input:    *k*: the number of error bounds
      *ε*[1…*k*]: the *k* error bounds used by *HBEPLA-RR*
      *BEPLA*[1…*k*]: *BEPLA*[*i*] is a BEPLA, *f_i_*(*x*), generated by *HBEPLA-RR*
      ε*: the required error bound
Output: *Y*’[1…*n*]: the approximated time series, which is
      *F_m_*(*x*) + *ε_m_* = *f*_1_(*x*) + *f*_2_(*x*) + … + *f_m_*(*x*) + *ε_m_*

1: **begin**2:   *Y*’[1…*n*] := 03:   **for** *m* := 1 to *k* **do**4:      *Y*’[1…*n*] := *Y*’[1…*n*] + ***Reconstruct*** (*BEPLA*[*m*])5:      **if** ε* ≥ *ε*[*m*] **then**6:         **break**7:      **end if**8:   **end for**9:   **return** *Y*’[1…*n*] + *ε*[*m*]10:  **end**


Based on the above mathematical proof, ***Approximate*** iteratively adds a BEPLA until the required error bound is guaranteed. The time and space complexities of ***Approximate*** are *O*(*kn*) and *O*(*n*), respectively. In a typical setting, *k* is small.

In practice, when a request comes, the storage system that keeps all BEPLAs decides how many BEPLAs to send back. Thus, a variant of ***Approximate*** is used in the requesting node, as it just has to sum up the returned BEPLAs. The complexity of the variant is thus *O*(*mn*), when *m* BEPLAs are received, 1 ≤ *m* ≤ *k*.

**Figure 5:**
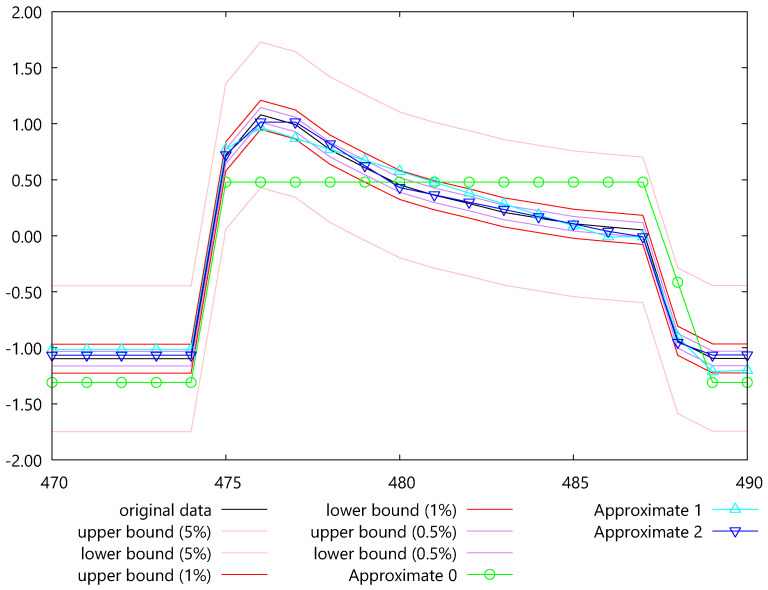
The three approximations for dataset Cricket_X w.r.t. the three error bounds generated by HBEPLA-RR.

Figure 5 shows the three time series reconstructed by the algorithm ***Approximate***. All three approximations are between their corresponding lower and upper bounds.

## 4. Experiment Results

In this section, real-world datasets are used to evaluate the performance of PBEPLA-RR. The UCR time series classification archive [1] is widely used for different purposes, including performance analysis in [8] for optimal disconnected BEPLA, refs. [10,11] for optimal mixed BEPLA, and [12] for Swing-RR. Eight datasets from the archive are chosen. They are Cricket_X, Cricket_Y, Cricket_Z, FaceFour, Lighting2, Lighting7, MoteStrain, and Wafer. The selection is the same as that in [12].

Three typical error bounds, 5%, 1%, and 0.5%, are used. Both connected and disconnected BEPLAs are generated with different maximal delay values.

Approximations for each error bound are generated independently by Swing-RR. Minimal resolution is used according to the relative error bound. The resolutions are 4, 6, and 7, respectively. On the other hand, in PBEPLA-RR, the actual relative error bounds for the three ***Swing-RR*** invocations are 5%, 8.3%, and 16.7%, and thus the corresponding resolutions are 4, 3, and 2, respectively. Again, minimal resolution is used. Table 2 shows the parameters used in Independent Swing-RR and PBEPLA-RR.

### 4.1. Connected and Disconnected PBEPLA-RR

**Figure 6:**
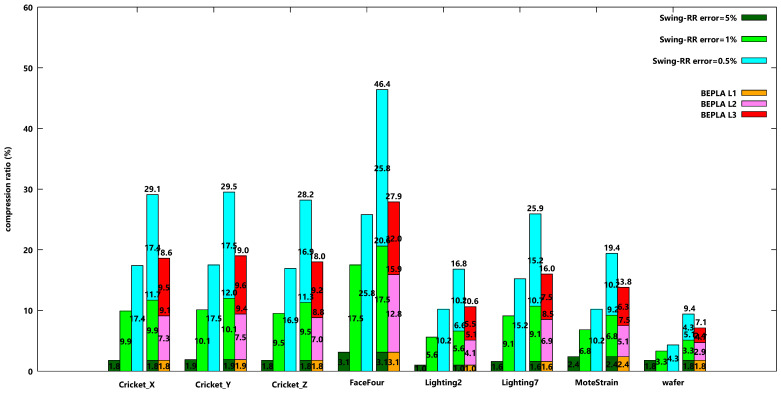
Compression ratios when connected Swing-RR are used to approximate all eight datasets w.r.t. different error bounds, 5%, 1%, and 0.5%, respectively, and connected PBEPLA-RR w.r.t. progressive error bounds, from 5% to 1% and further to 0.5%. The maximal delay is 64 time ticks.

Figure 6 shows the compression ratios of eight datasets when Swing-RR is used to find connected BEPLAs for each error bound independently and PBEPLA-RR is used to find connected BEPLAs for one error bound and progressively for another error bound until all error bounds are applied. The maximal delay is 64 time ticks. I.e., the maximal segment length is 64.

It is easy to see that the smaller the error bound, the larger the compression ratios when Swing-RR is used independently to generate a BEPLA for each error bound. For dataset Cricket_X, the compression ratios for error bounds 5%, 1%, and 0.5% are 1.8%, 9.9%, and 17.4%, respectively, when Swing-RR is used. When all three approximations (or versions) of the original data w.r.t. different error bounds are kept in the systems, the total data size accounts for 29.1% of the original data size.

On the other hand, the BEPLAs for the second and third error bounds generated by PBEPLA-RR use only 7.3% and 9.5% of the original data size. The total data size is 18.6%. To obtain a version w.r.t. 1% error bound, the data size is 9.1%, which is even smaller than the BEPLA independently generated by Swing-RR. To obtain a version with a 0.5% error bound, 18.6% is required, which is only 1.2% larger than the BEPLA independently generated by Swing-RR.

In summary, for dataset Cricket_X, without progressive bounded error approximation, the system may have two approaches. The first approach is to keep only the version w.r.t. the smallest error bound, 0.5%. It costs 17.4% of the original data size. However, when a request comes, the only version is returned, even if the requested error bound is 5% or 1%. The second is to generate all three versions of the original data w.r.t. 5%, 1%, and 0.5% error bounds, respectively. It costs 29.1% of the original data size. When a request comes with an error bound, the nearest version is returned. When the request error bound is 5% or 1%, the required network bandwidth is significantly reduced to 1.8 or 9.5%, respectively.

With the progressive bounded error approximation PBEPLA-RR used in this paper, it costs 18.6% of the original data size to store the three BEPLAs. That is significantly smaller than the second approach, 29.1%, and a tiny larger than the first approach, 17.4%. However, the required network bandwidth to transmit any version is comparable to the second approach, as shown in Table 3.

Table 3 also shows the ratio between the measured RMSE (Root Mean Square Error) of an approximation and its error bound. It reveals that for every approximation w.r.t. an error bound, the average difference between the approximation and the original time series is half of the error bound or so. There is no significant difference between the three approaches.

The behaviors for all other 7 datasets are similar to Cricket_X.

The authors note that except for MoteStrain and wafer, for the other 6 datasets, the versions with a 1% error bound are smaller than the corresponding versions independently generated by Swing-RR.

Resolution Reduction (RR) introduced in [12] plays an important role. Although the endpoints are restricted, and therefore Swing-RR uses more line segments to approximate the target time series. However, Swing-RR benefits a lot from the bit reduction, i.e., from 32-bit floating-point real numbers to small unsigned integers. Refer to Figure 4. Endpoint selection of line segments in transient periods is usually more restricted by the data itself.

**Figure 7:**
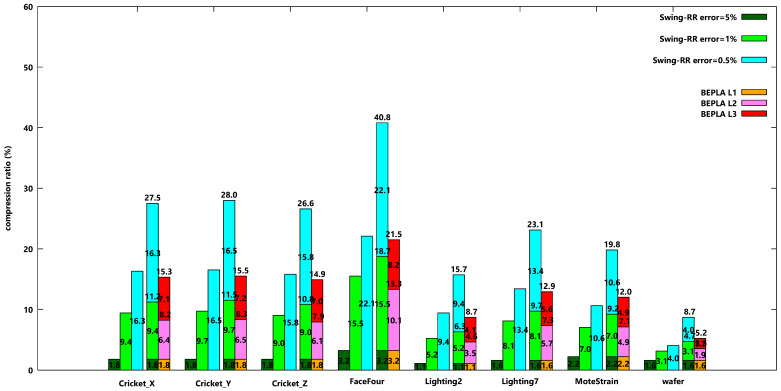
Compression ratios when disconnected Swing-RR are used to approximate all eight datasets w.r.t. different error bounds, 5%, 1%, and 0.5%, respectively, and disconnected PBEPLA-RR w.r.t. progressive error bounds, from 5% to 1%, and further to 0.5%. The maximal delay is 64 time ticks.

In addition to connected BEPLAs, scenarios for disconnected BEPLAs are also investigated. Figure 7 shows the compression ratios of eight datasets when a similar experiment is taken, except that disconnected BEPLAs are generated. The behaviors shown in Figure 7 are similar to Figure 6. However, the compression ratios of disconnected Swing-RR are consistently smaller than those of connected Swing-RR, and so are the compression ratios for disconnected HBEPLA-RR.

In fact, except for MoteStrain and wafer, for all other 6 datasets, the total costs used by HBEPLA-RR are even smaller than the approach that stores only one version w.r.t. the smallest error bound. Table 4 shows the experiment results for dataset Cricket_X. Also, the RMSE of all versions is given. The behavior is similar to that in connect PBEPLA-RR.

### 4.2. Maximal Delay

**Figure 8:**
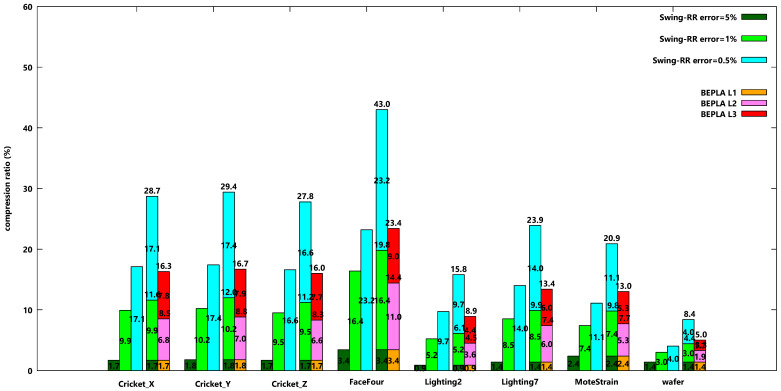
Compression ratios when disconnected Swing-RR are used to approximate all eight datasets w.r.t. different error bounds, 5%, 1%, and 0.5%, respectively, and disconnected PBEPLA-RR w.r.t. progressive error bounds, from 5% to 1% and further to 0.5%. The maximal delay is 128 time ticks.

**Figure 9:**
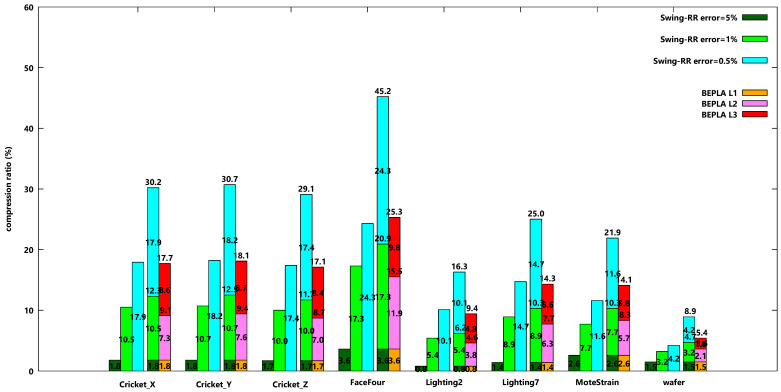
Compression ratios when disconnected Swing-RR are used to approximate all eight datasets w.r.t. different error bounds, 5%, 1%, and 0.5%, respectively, and disconnected PBEPLA-RR w.r.t. progressive error bounds, from 5% to 1%, and further to 0.5%. The maximal delay is 256 time ticks.

Figure 8 and Figure 9 show the compression ratios of eight datasets when similar experiments are taken, except that different maximal delays are used. When maximal delay increases, some longer line segments are generated. However, the bits used to encode the segment length also increase. The experiment results show that the compression ratios do not further decrease when maximal delay increases.

Here, only the experiment results of disconnected Swing-RR and PBEPLA-RR are shown. However, the behaviors for connected Swing-RR and PBEPLA-RR are similar.

### 4.3. Summary and Discussion

The experiment results show as follows:

- Disconnected PBEPLA-RR consistently outperforms connected PBEPLA-RR on all eight datasets.
- For all datasets, enlarging the maximal delay from 64 time ticks to 128, and further to 256, does not reduce the compression ratios.
- The total cost of HBEPLA-RR is comparable to that of the first approach that stores only one version w.r.t. the smallest error bound. In fact, except for MoteStrain and wafer, for all other 6 datasets, the total cost of disconnected HBEPLA-RR is even smaller than the first approach. For MoteStrain and water, the difference in total cost between the two approaches is 1% or so of the original data size.
- For all datasets, the total cost of HBEPLA-RR is significantly smaller than that of the second approach that keeps multiple independent versions.
- The costs of most versions generated by HBEPLA-RR are comparable to those of their corresponding versions generated independently by Swing-RR. In fact, except for MoteStrain and wafer, for all other 6 datasets, the versions w.r.t. error bounds of 1% and 0.5% generated by disconnected HBEPLA-RR are even smaller than their corresponding versions generated independently by Swing-RR.

Although the compression ratio is dependent more on the time series itself, HBEPLA-RR can effectively compress the series and provides multiple compressed versions w.r.t. different error bounds.

## 5. Conclusions

Given an error bound, Bounded Error Piecewise Linear Approximations (BEPLAs) are commonly used to reduce the data size of a time series. However, different applications in different scenarios may have different data accuracy requirements. In general, there are three approaches. The first is to keep just one version w.r.t. the smallest error bound. Trivially, the only one version is always returned, no matter what the required error bound is. The second is to keep multiple approximations (or versions), each of which is w.r.t. a specific error bound. When a request comes, the nearest version is returned. And the third is to adopt progressive bounded error approximation.

In this article, a novel progressive bounded error approximation algorithm is proposed, referred to as PBEPLA-RR. Given *k* different error bounds, PBEPLA-RR generates *k* BEPLAs by invoking Swing-RR *k* times. However, the parameters for each Swing-RR invocation are adjusted for progressive bounded error approximation, especially the relative error bounds. As a result, Resolution Reduction (RR) plays a significant role in data compression. Swing-RR is simple and effective to find a BEPLA. Its time and space complexities are *O*(*n*) and *O*(*d*), where *n* and *d* are the number of data points and maximal delay, respectively. As a result, the time complexity of HBEPLA-RR is *O*(*kn*). With proper implementation, the space complexity remains *O*(*d*). This makes HBEPLA-RR simple and suitable to be adopted in sensor nodes and edge nodes.

Eight real-word datasets, selected from the UCR time series classification archive, are used to evaluate the performance of HBEPLA-RR. Three common error bounds are specified. They are 5%, 1%, and 0.5%. In the beginning, three versions are generated independently by Swing-RR w.r.t. each error bound. For the first approach, the total cost is the size of the version w.r.t. the smallest error bound. For the second approach, the total cost is the total size of the three versions. Then, three components are generated by PBEPLA-RR. Another three versions are synthesized by adding up the three components incrementally. For the third approach, the total cost is the total size of the three components.

The experiment results show that although the compression ratios are somehow dependent on the time series itself, disconnected HBEPLA-RR consistently outperforms connected HBEPLA-RR. The total size that HBEPLA-RR uses is almost the same as the size that the first approach uses and significantly smaller than the size that the second approach uses. Furthermore, the sizes of versions generated by disconnected HBEPLA-RR are comparable to the sizes of their corresponding versions kept by the second approach. As a result, PBEPLA-RR can achieve very good compression ratios and provide multiple compressed approximations w.r.t. different error bounds.

The authors note that the components generated by HBEPLA-RR can be further compressed by lossless data compression techniques, such as entropy encoding and run-length encoding. Although Swing-RR is simple and effective, it is not optimal in terms of compression ratios under the concept of BEPLA with RR. This implies that HBEPLA-RR is not optimal. In addition, it had been reported that mixed BEPLA algorithms constantly outperform both connected and disconnected BEPLA algorithms. Optimal algorithms for BEPLA with RR are under investigation.

## Figures and Tables

**Table 1:** Minimal resolutions for common relative error bounds.

Error bound (%)	0.1	0.2	0.3	0.4	0.5	1	2	3	4	5	10	20
Minimal resolution (bits)	9	8	8	7	7	6	5	5	4	4	3	2

**Table 2:** Parameters used in Swing-RR and PBEPLA-RR.

Desired Relative Error Bound	Independent Swing-RR		PBEPLA-RR	
	Relative Error Bound	Resolution	Actual Relative Error Bound	Resolution
Error bound 1 = 5%	5%	4	5%	4
Error bound 2 = 1%	1%	6	8.3% = 1%/(2 × (1% + 5%))	3
Error bound 3 = 0.5%	0.5%	7	16.7% = 0.5%/(2 × (0.5% + 1%))	2

**Table 3:** Comparison of three approaches to compress Cricket_X and then to retrieve versions w.r.t. different error bounds. Connect BEPLAs are used.

	One Version w.r.t. the Smallest Error Bound	Three Versions Generated Independently	PBELPA-RR
Total cost	17.4%	29.1%	18.6%
Cost of version 1 (5%)	–	1.8%	1.8%
Cost of version 2 (1%)	–	9.9%	9.1% = 1.8% + 7.3%
Cost of version 3 (0.5%)	17.4%	17.4%	18.6% = 9.1% + 9.5%
RMSE of version 1 (5%)	–	46.5%	46.5%
RMSE of version 2 (1%)	–	51.8%	51.9%
RMSE of version 3 (0.5%)	52.9%	52.9%	54.3%

**Table 4:** Comparison of three approaches to compress Cricket_X and then retrieve versions with different error bounds. Disconnect BEPLAs are used.

	One Version w.r.t. the Smallest Error Bound	Three Versions Generated Independently	PBELPA-RR
Total cost	16.3%	27.5%	15.3%
Cost of version 1 (5%)	–	1.8%	1.8%
Cost of version 2 (1%)	–	9.4%	8.2% = 1.8% + 6.4%
Cost of version 3 (0.1%)	16.3%	16.3%	15.3% = 8.2% + 7.1%
RMSE of version 1 (5%)	–	44.1%	44.1%
RMSE of version 2 (1%)	–	49.0%	48.9%
RMSE of version 3 (0.5%)	50.5%	50.5%	50.6%

## Data Availability

The UCR time series classification archive is available online [1].

## Abbreviations

AI	Artificial Intelligent
AIoT	Artificial Intelligent + Internet of Things
BEPLA	Bounded-Error Piecewise Linear Approximation
HIRE	Hierarchical Residual Encoding
IoT	Internet of Things
PBEPLA-RR	Progressive BEPLA with Resolution Reduction
RMSE	Root Mean Square Error
RR	Resolution Reduction
Swing-RR	Swing Filter with Resolution Reduction
VBR	Variable Bit Rate
